# Application of droplet digital polymerase chain reaction of plasma methylated septin 9 on detection and early monitoring of colorectal cancer

**DOI:** 10.1038/s41598-021-02879-8

**Published:** 2021-12-06

**Authors:** Zhi Yao Ma, Cherry Sze Yan Chan, Kam Shing Lau, Lui Ng, Yuen Yee Cheng, Wai K. Leung

**Affiliations:** 1 Department of Medicine, Queen Mary Hospital, University of Hong Kong, Hong Kong, China; 2 Department of Surgery, Queen Mary Hospital, University of Hong Kong, Hong Kong, China; 3 Asbestos Diseases Research Institute, Sydney Medical School, The University of Sydney, Sydney, Australia

**Keywords:** Cancer epigenetics, Biomarkers, Colorectal cancer

## Abstract

Methylated septin 9 (SEPT9) has been approved for non-invasive screening of colorectal cancer (CRC), but data on monitoring of CRC is sparse. Droplet digital polymerase chain reaction (ddPCR), with higher detection precision and simpler quantification than conventional PCR, has not been applied in SEPT9 detection. We explored the role of SEPT9 ddPCR for CRC detection and to measure serial SEPT9 levels in blood samples of CRC patients before and 3-month after surgery. SEPT9 methylated ratio, methylated abundance, and CEA levels were all higher in CRC patients than normal controls (all P < 0.05). The area under the curve (AUC) for methylated ratio and abundance to detect CRC was 0.707 and 0.710, respectively. There was an increasing trend for SEPT9 methylated abundance from proximal to distal cancers (P = 0.017). At 3-month after surgery, both methylated abundance and ratio decreased (P = 0.005 and 0.053, respectively), especially methylated abundance in stage III and distal cancer (both P < 0.01). We have developed a ddPCR platform for the quantitative detection of plasma SEPT9 in CRC patients. SEPT9 methylated abundance had an early post-operative decline, which may be useful in monitoring of treatment response.

## Introduction

Colorectal cancer (CRC) has emerged as the commonest cancer in Hong Kong since 2013. The crude incidence rate in men and women was 97.4 and 58.3 per 100,000 in 2017, respectively^1^. Due to the ageing population, the incidence of CRC is predicted to continue to increase in many developed and developing regions^2^. While screening of CRC plays a pivotal role in reducing the incidence and mortality of CRC, non-invasive blood test is still favored by many due to its simplicity and convenience. Additionally, for monitoring of CRC recurrence after curative treatment, a non-invasive blood test would be particularly preferred due to the need for repetitive measurements during follow-up.

Carcinoembryonic antigen (CEA) is a commonly used blood marker that has been recommended by the American Society of Clinical Oncology and European Group on Tumor Markers for monitoring CRC patients. However, a review analysis including 52 studies showed unsatisfactory results^3–5^. In addition to the blood glycoprotein marker, circulating molecular biomarkers, including microsatellites loci, RNA markers, and methylation biomarkers, could be generated by cancer cells or normal cells during carcinogenesis and detected in blood for cancer diagnosis, prognosis, and treatment response^6,7^. Among various epigenetic biomarkers with characteristics of stability and repeatability, DNA methylated biomarkers have been studied most frequently^8–10^.

Septin 9 (SEPT9), which belongs to the septin family and is involved in cytokinesis and cell cycle control, has been studied in many cancers including ovarian cancer, lung cancer, and CRC^11–14^. As a tumor suppressor gene, the higher methylated level of septin 9 could inhibit gene expression and promote cancer progression^15^. Among various DNA methylated biomarkers, methylated SEPT9 in blood is the only one approved by the Food and Drug Administration (FDA) of the United States as a non-invasive method for CRC screening and has been evaluated in several studies on CRC diagnosis^16–20^. Recently, we have also explored the possibility of using SEPT9 in the monitoring of CRC patients after surgery^21^. As yet, the currently available test is qualitative and its role in quantitative monitoring of CRC is less useful.

Methods used to detect DNA methylation are usually based on polymerase chain reaction (PCR)^22^. Droplet digital PCR (ddPCR), a recently emerged quantitative PCR technology, has been applied in methylation-related cancer studies including lung cancer and CRC. ddPCR could provide greater accuracy and simpler quantification than conventional qPCR^23–26^. The commonly used Bio-Rad ddPCR system could distribute target samples into 20,000 discrete water-in-oil droplets and calculate the inside nucleic acid molecules, allowing a smaller amount of template DNA than conventional real-time qPCR^27^.

In this study, we evaluated the feasibility and performance of our newly developed ddPCR in detecting plasma methylated SEPT9 levels in CRC patients and monitoring the quantitative changes of SEPT9 methylation levels in the early post-operative period after curative surgery. Further comparison with CEA was also performed.

## Method

### Participants

Study subjects were prospectively recruited from the Queen Mary Hospital in Hong Kong, which is a major public regional hospital. A total of 103 CRC patients and 32 normal controls were enrolled in the study. All CRC patients were confirmed to have adenocarcinoma and planned for curative resection of the primary tumor. Control subjects were identified from those who underwent colonoscopy in the Endoscopy Center of the Hospital for screening, bowel symptoms, or diagnostic workup of iron deficiency anemia, and had normal colonoscopy. We excluded patients with previous bowel resection, familial colorectal cancer syndrome, inflammatory bowel disease, or other malignancy in the past. Tumor staging was classified according to the 7th edition of the Cancer Staging Manual of the American Joint Committee on Cancer^28^.

Blood samples were taken immediately before surgery in CRC patients or before colonoscopy in control subjects, and then every about 3 months during follow-up from post-operative cancer patients. During the follow-up, local recurrence and new metastases (including lymph nodes and distant metastasis) were monitored for CRC patients through CT scan or PET-CT scan. Surveillance colonoscopy was also performed according to usual clinical guidelines. Plasma was stored at −70 °C till further processing after separation from blood cells through 15 min’ centrifugation with 3000 revolutions per minute, 8 acceleration level, and 9 deceleration level at 25 °C room temperature. For early monitoring, paired plasma samples from cancer patients before operation and at 3-month after operation were selected for further ddPCR test.

The study protocol was approved by the Institutional Review Board of the Hospital Authority-Hong Kong West Cluster and University of Hong Kong (UW 12-489). Written informed consent was obtained from all participants. The study was conducted according to the criteria set by the declaration of Helsinki and local regulatory requirements.

### DNA extraction and bisulfite conversion

Genomic DNA was extracted from stored plasma samples through QIAamp^®^ DNA Mini Kit (Qiagen, Venlo, Netherlands). A total of 25 μL DNA were obtained from 250 μL plasma through water bath incubation, mixing, and centrifugation. After DNA extraction, 22.5 μL plasma DNA was treated immediately with the reagents in the EZ DNA Methylation™ Kit (Zymo Research, California, U.S.). After several times of mixing and centrifugation, and twice water bath, including 15 min at 37 ºC after adding the M-Dilution Buffer and 12–16 h dark incubation at 50 ºC after adding the CT-Conversion Reagent, 10 μL bisulfite DNA was obtained finally. Through the methylation kit, methylated cytosine remained the same while most unmethylated cytosine residues were converted into uracil and recognized as thymine in following PCR amplification^29^.

As controls, DNA from ten CRC cell lines (SW480, DLD1, HT29, HCT116, Colo205, Colo320, LoVo, LS123, SW1116, and HCT15) and two cancer-adjacent normal colon tissues (labeled as 265NC and 758NC), which were obtained from the Department of Surgery, the University of Hong Kong, were used for validation of SEPT9 primers and probes used in methylation detection through ddPCR after bisulfite treatment. Unlike plasma DNA which had equal initial plasma volume, DNA in the cell lines and tissues was adjusted to the same DNA concentration for validation in ddPCR.

### Droplet digital PCR for SEPT9 detecting

To ensure the quality was not affected by freezing, plasma SEPT9 detection was conducted immediately after bisulfite treatment through QX200™ ddPCR System (Bio-Rad, California, U.S.). The total volume of the ddPCR reaction mixture was 25 μL, consisting of 8 μL modified plasma DNA, 12.5 μL 1 × ddPCR Supermix for Probes without dUTP (Bio-Rad), primers and probes with the final concentration of 800 nM and 400 nM, respectively. Sequences of forward and reverse primers were adapted from previous study: 5’-AGAGAATTTTGTTTGGTTGTTTAAATATAG-3’ and 5’-AAAAAAAATTCCTCCCCTTCC-3’^30^. The methylated and unmethylated probes were designed through Oligo Primer Analysis Software version 7 (Oligo, Colorado, U.S.), and the sequences were 5’-/56-FAM/TGTAGAAGG/ZEN/ATTTTGCGTTCGG/3IABkFQ/-3’ and 5’-/5HEX/TTGTAGAAG/ZEN/GATTTTGTGTTTGG/3IABkFQ/-3’ (Supplementary Fig. S1). Each probe was designed with a fluorescent reporter dye 6-FAM or HEX at 5’ end, an internal ZEN Quencher, and a Black Quencher dye IABkFQ at 3’ end. All primers and probes were purchased from Integrated DNA Technologies (IDTA; Coralville, U.S.).

In each DG8™ Cartridges for QX100™/QX200™ Droplet Generator (Bio-Rad), eight prepared ddPCR mixtures (20 μL) were added to generate droplets with 70 μL Droplet Generation Oil for Probes (Bio-Rad) by QX200™ Droplet Generator (Bio-Rad). For each reaction mixture, there was about 40 μL generated liquid for following PCR amplification in C100 Touch™ Thermal Cycler (Bio-Rad) and droplet reading through the QX200™ Droplet Reader (Bio-Rad) according to the protocol^27^. After the initial 95 ºC for 10 min, there were 40 cycles including 94 ºC for 30 s and 52 ºC for 1 min in the PCR amplification, with a final 98 ºC for 10 min and keeping temperature of 4 ºC. For each ddPCR cycling, there were a negative non-template control (NTC; water) and a positive control by using the HCT116 cell line with methylated SEPT9. All PCR reactions were repeated in triplicates.

### CEA measurement

CEA levels were determined by enzyme-linked immunoassay in the Clinical Immunology Laboratory of the hospital. Abnormal CEA was defined as a value above 3 ng/ml as recommended by the laboratory.

### Data presentation and analysis

SEPT9 methylation data obtained from the QX200™ Droplet Reader (Bio-Rad) was analyzed in QuantaSoft Analysis Pro Software version 1.0 (Bio-Rad). In the amplitude graph, grey dot indicated negative while blue and green dot indicated positive for Fam- and Hex-labelled probes after defining thresholds, respectively. Methylated and unmethylated SEPT9 copies in 20 μL reaction and corresponding concentration (copies per μL) were shown according to the fraction of positive droplets. The methylated ratio was defined as the methylated to unmethylated concentration/copies whereas the methylated abundance referred to the fraction of methylated concentration/copies in total. The methylation results and patients’ clinicopathologic characteristics were analyzed by the SPSS Software version 21 (IBM, New York, U.S.) and the GraphPad Prism version 7 (GraphPad Software, San Diego, CA), with 95% confidence intervals (CIs) and a P value of less than 0.05 was considered statistically significant.

## Results

### Validation of SEPT9 in CRC cell lines and normal tissues by ddPCR

The SEPT9 methylation in CRC cell lines and cancer-adjacent normal tissues through ddPCR was shown in Fig. S2, including the tenfold serial dulition assay for HCT116 cell line. Higher methylated ratio and abundance were shown in eight CRC cell lines, except SW480 and HT29 as well as the two normal tissues (mean ratio 8.2 *versus* 0.02, P = 0.013; mean abundance 66.6% versus 1.88%, P < 0.001). The highest methylated ratio was detected in LoVo, followed by HCT116 and SW1116.

### Diagnostic value of SEPT9 ddPCR for CRC

After validation of the SEPT9 ddPCR protocol, we measured the SEPT9 methylation levels in 103 CRC patients (male 61.2%; age 67.3 ± 10.3 years) and 32 normal subjects (male 37.5%; age 63.2 ± 5.9 years). There were 27, 37, 33, and 3 CRC patients with stage I to IV cancer, respectively whereas three cases had unknown staging (Table 1).

**Table 1:** Basic characteristics of patients and control subjects for SEPT9 detection by ddPCR.

Subjects	No. of cases	Female (%)	Age ± SD in years
CRC	103	38.8	67.3 ± 10.3
Stage I	27	37.0	68.2 ± 9.8
Stage II	37	32.4	69.3 ± 10.4
Stage III	33	42.4	64.9 ± 10.6
Stage IV	3	66.7	67.0 ± 6.6
Unknown Staging	3	66.7	62.3 ± 10.4
Normal Control	32	62.5	63.2 ± 5.9

*SEPT9* septin 9, *ddPCR* droplet digital polymerase chain reaction, *SD* standard deviation, *CRC* colorectal cancer.

When compared to normal controls, CRC patients had higher SEPT9 methylated concentration (1.47 versus 0.78, P = 0.06), methylated ratio (1.27 versus 0.04, P = 0.02) and methylated abundance (27.1% versus 3.97%, P < 0.001). CEA levels were also higher in CRC patients than normal controls (6.1 versus 1.7, P = 0.004).

**Figure 1:**
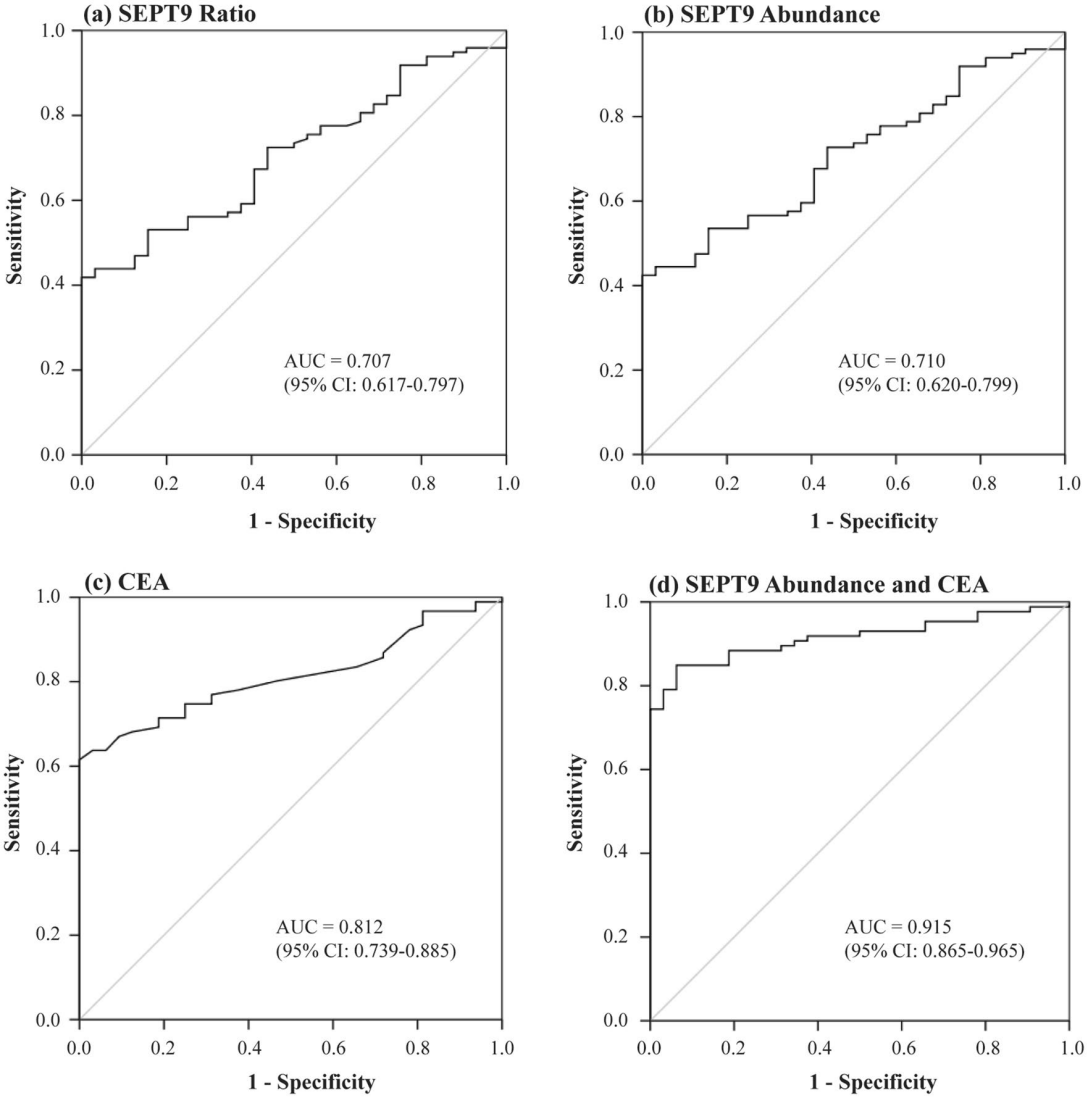
The ROC curve for detecting CRC by SEPT9 methylation and CEA. **(a,b)** showed the receiver operating characteristic (ROC) curves for septin 9 (SEPT9) methylated ratio and abundance to distinguish colorectal cancer (CRC) patients from normal subjects; **(c)** the ROC curve for carcinoembryonic antigen (CEA); and **(d)** the ROC curve of combining CEA and SEPT9 methylated abundance.

Figure 1 showed the receiver operating characteristic (ROC) curves for the corresponding SEPT9 methylation levels to distinguish CRC patients from normal subjects. The area under the ROC curve (AUC) for SEPT9 methylated ratio was 0.71 (95% CI: 0.62–0.80) and SEPT9 abundance was AUC 0.71 (95% CI: 0.62–0.80), but was only 0.55 (95% CI: 0.46–0.64) for methylated concentration. The optimal cut-off value for SEPT9 methylated ratio to detect CRC was 0.03, while for methylated abundance was 2.95%. With these cut-off values, the specificities were both 50% with corresponding sensitivities of 73.5% and 73.7% for ratio and abundance, respectively. When increasing the cut-off values of the ratio to 0.14 and abundance to 12.3%, 100% specificity could be obtained but at lower sensitivities of 41.8% and 42.4%, respectively.

When combining individual clinical parameters including patient’s age and sex, CEA values, and SEPT9 methylated results of concentration, ratio, or abundance to determine the presence of CRC, the combination of SEPT9 methylated abundance and CEA was found to be the best in detecting CRC. This combination of them could produce an AUC of 0.92, which was higher than either marker alone (0.71 and 0.81, respectively, Fig. 1D).

**Figure 2:**
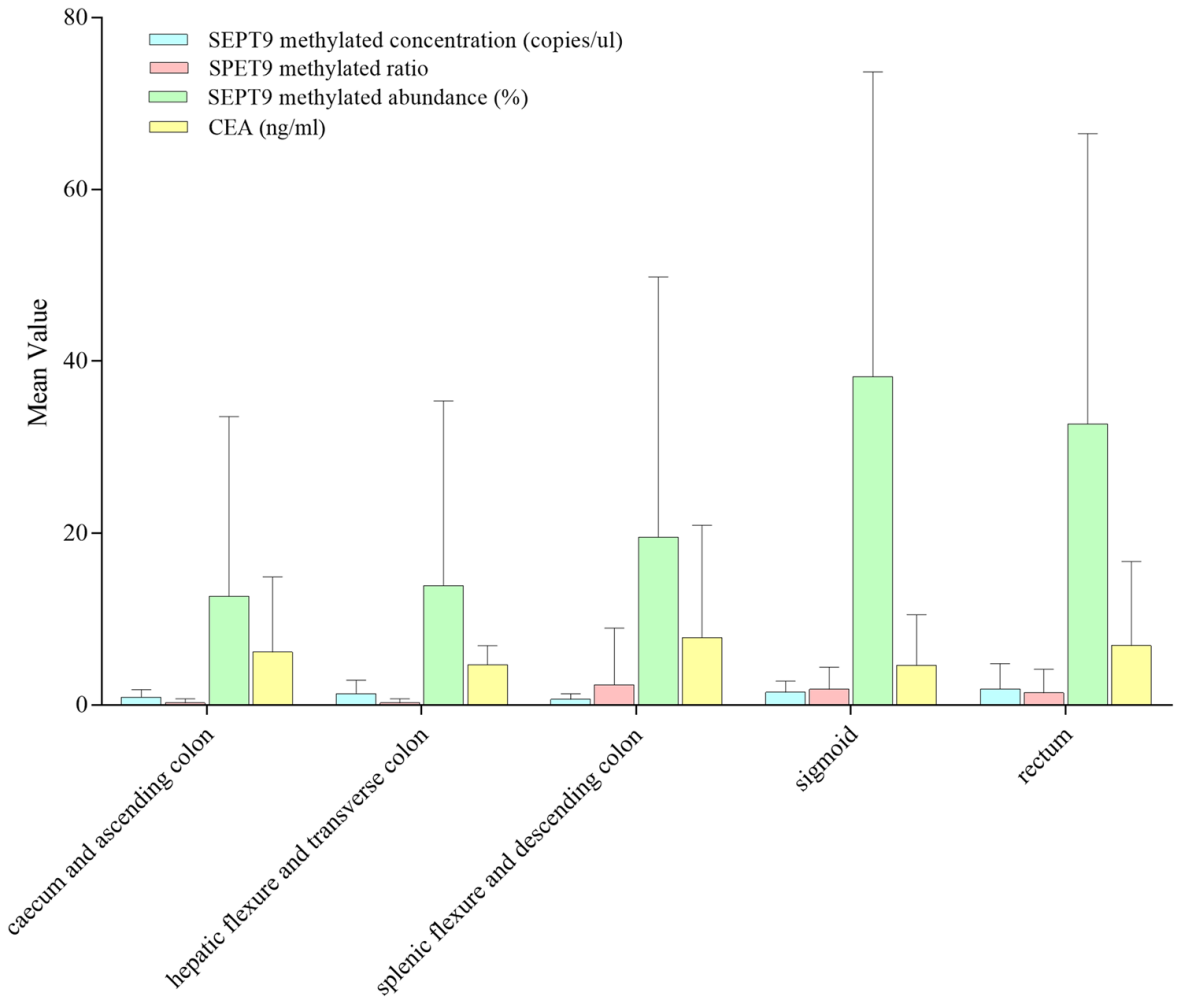
SEPT9 methylation and CEA levels in patients according to tumor locations. The mean values of pre-operative septin 9 (SEPT9) methylated concentration (blue), ratio (pink), abundance (green), and carcinoembryonic antigen (CEA, yellow) levels were shown for colorectal cancer (CRC) patients according to tumor locations from the caecum and ascending colon to the rectum. The methylated abundance increased from proximal to distal sites (Pearson Correlation Coefficient 0.25, P = 0.017) while SEPT9 methylated concentration, ratio and CEA had no such significant trend (Pearson Correlation Coefficient 0.16, 0.13 and 0.03; P = 0.11, 0.21 and 0.76, respectively).

In subgroup analysis, patients with distal cancer had higher SEPT9 methylated abundance than those with proximal cancer (31.4% *versus* 12.7%, P = 0.017). Specifically, the methylated abundance progressively increased from proximal to distal sites (caecum and ascending colon 12.7%; hepatic flexure and transverse colon 13.9%, splenic flexure and descending colon 19.5%; sigmoid 38.2%, and rectum 32.7%; Pearson Correlation Coefficient 0.25, P = 0.017; Fig. 2). There was no association between tumor location and SEPT9 methylated concentration, ratio, or CEA. Moreover, there was no significant association between SEPT9 methylated levels or CEA and tumor stages or patients’ characteristics.

### Early post-operative monitoring by SEPT9 ddPCR in CRC patients

**Figure 3:**
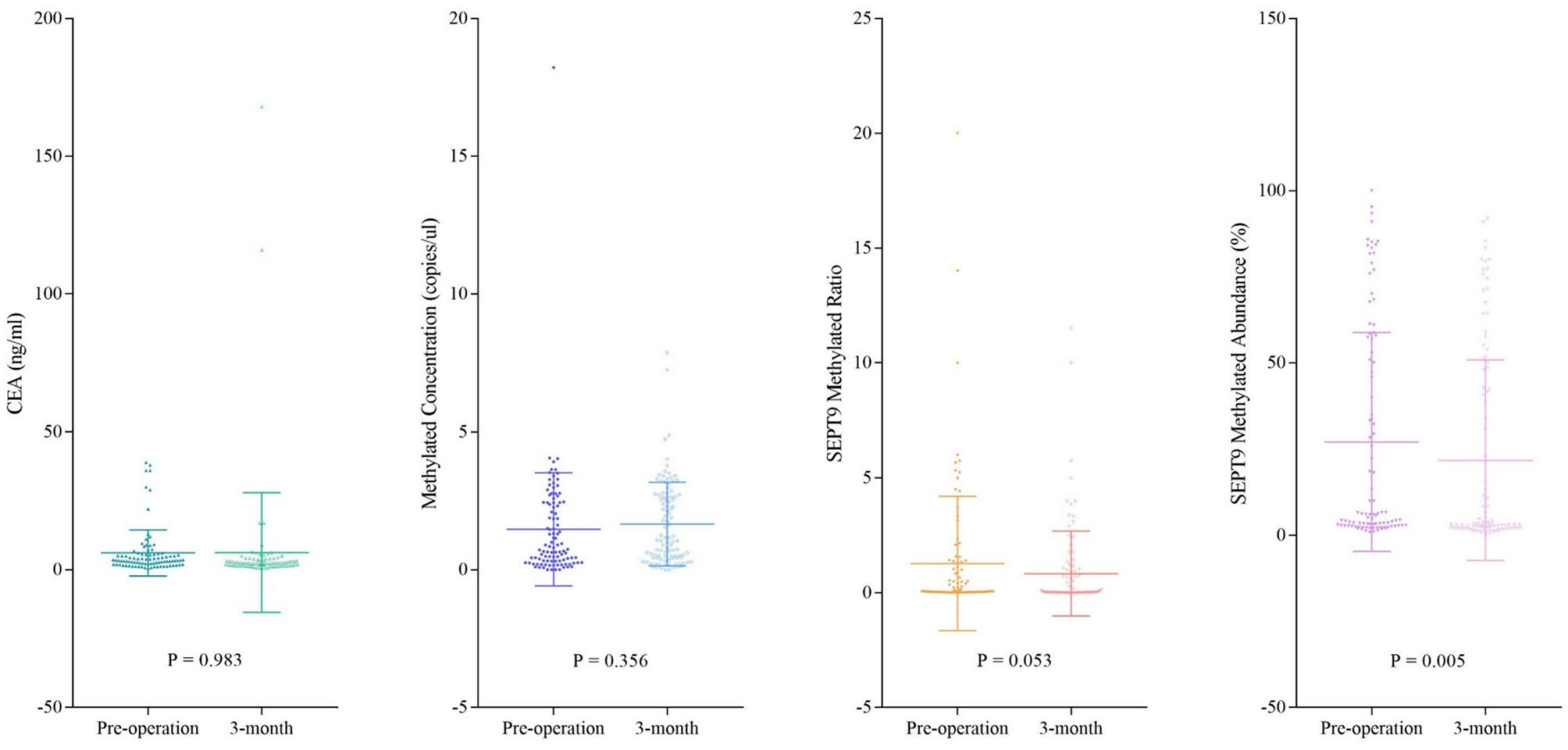
Changes in CEA and SEPT9 methylation levels at 3-month post-operation. This figure showed the changes of SEPT9 methylated status and CEA levels at 3-month after surgery.

The changes in SEPT9 methylated status and CEA levels at 3-month after curative surgery for CRC were shown in Fig. 3. There was a significant decrease in SEPT9 methylated abundance from pre-operation to 3-month post-operation (27.0% versus 22.6%, P = 0.005), but not for SEPT9 methylated ratio (1.28 versus 0.77, P = 0.053) or CEA levels (6.17 versus 6.22, P = 0.98).

Subgroup analysis showed that in patients with distal cancer, both the methylated abundance and ratio had significant reduction by 5.77% (P = 0.003) and 0.68 (P = 0.04) at 3-month, respectively (Table 2). When considering different tumor staging, stage III cancer had a more significant drop of the methylated abundance (P = 0.001), especially for distal CRC (P < 0.001). In contrast, CEA levels decreased significantly in patients with stage I cancer at 3-month (P = 0.04).

**Table 2:** SEPT9 methylation and CEA levels in CRC patients pre- and 3-month post-operation.

CRC Groups	Mean value of methylated concentration (copies/μL)			Mean value of methylated ratio			Mean value of methylated abundance (%)			Mean value of CEA (ng/ml)		
	Pre-operation	3-month	P-value	Pre-operation	3-month	P-value	Pre-operation	3-month	P-value	Pre-operation	3-month	P-value
All cases	1.47	1.66	0.36	1.28	0.77	0.05	26.98	22.57	**0.005**	6.17	6.22	0.98
Female	1.73	1.64	0.76	1.62	0.97	0.11	30.62	26.43	0.06	6.11	8.67	0.50
Male	1.06	1.69	**0.03**	0.69	0.43	0.07	20.97	16.22	**0.02**	6.27	2.55	**0.01**
Stage I	0.86	1.49	0.07	0.38	0.30	0.52	15.98	14.18	0.45	3.43	2.51	**0.04**
Stage II	1.57	1.62	0.74	1.54	1.27	0.49	34.89	31.69	0.34	7.15	8.69	0.81
Stage III	1.67	1.74	0.89	1.12	0.43	0.24	19.61	14.75	**0.001**	7.95	7.34	0.86
Stage IV	2.24	2.07	0.41	2.59	1.94	0.58	53.12	48.62	0.56	4.10	4.85	0.80
Proximal^a^	1.06	2.02	**0.049**	0.25	0.32	0.54	12.74	13.00	0.88	6.23	2.83	0.05
Distal	1.59	1.57	0.94	1.58	0.90	**0.04**	31.24	25.47	**0.003**	6.18	7.04	0.77
Sigmoid	1.51	1.93	0.12	1.84	0.88	**0.02**	38.21	27.07	**0.001**	4.26	3.35	0.58
Rectum	1.87	1.41	0.28	1.45	1.02	0.32	32.48	26.81	**0.04**	7.04	10.69	0.51

Significant P-values are in bold.

*SEPT9* septin 9, *CEA* carcinoembryonic antigen, *CRC* colorectal cancer.

^a^Proximal referred to cancer above the splenic flexure.

Table 3 showed the proportion of patients with decrease in SEPT9 methylated values (concentration, ratio, and abundance) or CEA levels at 3-month post-operation. The proportion of all CRC patients with reduction in methylated ratio, abundance, and CEA was 67.0%, 67.3%, and 69.4%, respectively. Of all the SEPT9 values and CEA, SEPT9 methylated concentration had the lowest (39.8%) proportion of patients with reduced concentration in the post-operative samples. Subgroup analysis showed that more patients with sigmoid cancer had reduction in SEPT9 ratio (84.0%) and abundance (84.0) whereas a higher proportion (87.5%) of patients with proximal cancer had a drop in CEA.

**Table 3:** The proportion of patients with reduction in SEPT9 methylation and CEA levels 3-month post-operation.

CRC groups	Proportion of patients with reduction in				P-value (Chi-square test, 2-sided)					
	SEPT9 methylated concentration	SEPT9 methylated ratio	SEPT9 methylated abundance	CEA	Concentration versus ratio	Concentration versus abundance	Concentration versus CEA	Ratio versus abundance	Ratio versus CEA	Abundance versus CEA
All	**39.8% (41/103)**	67.0% (65/97)	67.3% (66/98)	69.4% (59/85)	** < 0.001**	** < 0.001**	** < 0.001**	1.0	0.75	0.87
Male	44.4% (28/63)	63.9% (39/61)	63.9% (39/61)	68.6% (35/51)	**0.03**	**0.03**	**0.01**	1.0	0.69	0.69
Female	32.5% (13/40)	72.2% (26/36)	73.0% (27/37)	70.6% (24/34)	**0.001**	**0.001**	**0.002**	1.0	1.0	1.0
Stage I	33.3% (9/27)	48.0% (12/25)	48.0% (12/25)	65.2% (15/23)	0.40	0.40	**0.046**	1.0	0.26	0.26
Stage II	45.9% (17/37)	72.2% (26/36)	72.2% (26/36)	75.0% (21/28)	**0.03**	**0.03**	**0.02**	1.0	1.0	1.0
Stage III	30.3% (10/33)	76.7% (23/30)	77.4% (24/31)	72.4% (21/29)	** < 0.001**	** < 0.001**	**0.002**	1.0	0.77	0.77
Stage IV	66.7% (2/3)	33.3% (1/3)	33.3% (1/3)	50.0% (1/2)	1.0	1.0	1.0	1.0	1.0	1.0
Proximal^a^	36.4% (8/22)	57.1% (12/21)	57.1% (12/21)	87.5% (14/16)	0.23	0.23	**0.002**	1.0	0.07	0.07
Distal	41.3% (33/80)	70.7% (53/75)	71.1% (54/76)	66.2% (45/68)	** < 0.001**	** < 0.001**	**0.003**	1.0	0.59	0.59
Sigmoid	38.5% (10/26)	**84.0% (21/25)**	**84.0% (21/25)**	**47.4% (9/19)**	**0.001**	**0.001**	0.76	1.0	**0.02**	**0.02**
Rectum	48.7% (19/39)	62.9% (22/35)	63.9% (23/36)	74.3% (26/35)	0.25	0.25	**0.03**	1.0	0.44	0.44

Significant values are in bold.

*SEPT9* septin 9, *CEA* carcinoembryonic antigen, *CRC* colorectal cancer.

^a^Proximal referred to cancer above the splenic flexure.

## Discussion

Although SEPT9 has been used in the screening and diagnosis of CRC, this is the first study to evaluate the application of quantitative ddPCR for SEPT9 detection in CRC patients. We first validated the ddPCR in ten CRC cell lines, in which some have been tested in previous studies^15,24,31^. Specifically, Pharo et al. detected single methylated concentration of SEPT9 and CDO1 in several CRC cell lines through ddPCR and observed that Colo205, Colo320, and HCT116 had higher SEPT9 methylated concentration than the other cell lines, with the lowest level in SW480, which was in accordance with our observation^24^. To ensure the validity of our findings, we have included HCT116 cell line as the positive control and negative control for each ddPCR reaction of the clinical samples in our study^32–34^. When determining the methylated SEPT9 levels by ddPCR, different ways of expressing the methylated levels have been used including methylated concentration, abundance, and ratio^24,35–37^. However, there is no gold standard so far. By using one methylated probe, only single methylated concentration or copies could be used to express the methylation status. With the additional unmethylated probe, the methylated ratio and abundance could be determined. In our study, SEPT9 methylated abundance or ratio was found to be better than single methylated concentration. The accuracy in terms of AUC of the methylated ratio and abundance on detecting CRC were both about 0.71, which was superior to the unsatisfactory performance of single methylated concentration (AUC 0.55). The best sensitivity in detecting CRC was about 74% with 50% specificity for methylated abundance. Notably, the combination of SEPT9 methylated abundance and CEA was found to improve the performance with AUC up to 0.92.

When determining the correlation between CRC clinicopathological characteristics and SEPT9 methylated levels, we found that the SEPT9 methylated abundance was significantly higher in distal cancer, especially in sigmoid cancer. A similar trend of the higher sensitivity of SEPT9 methylation in left-sided CRC has been previously reported in some studies^38–40^. In contrast, Xie et al. observed the highest sensitivity of SEPT9 in ascending colon (75.0%, N = 8) and the second highest level in the sigmoid colon (72.7%, N = 33)^41^. However, all these studies were based on conventional PCR rather than ddPCR.

In this study, we also evaluated the role of ddPCR in the monitoring of SEPT9 methylation changes in the early post-operative phase (i.e. 3-month after curative surgery). Of various SEPT9 expression levels, SEPT9 methylated abundance appeared to have the best early response post-operatively, with a significant reduction in SEPT9 methylated abundance at 3-month after surgery (from 27.0 to 22.6%), especially in distal cancer. When considering different tumor stages, stage III CRC had the most decline in SEPT9 methylated abundance after surgery. In contrast, CEA appeared to have a greater reduction in proximal cancer as well as in stage I cancer.

Thus far, few studies have evaluated the early changes in SEPT9 after curative surgery for CRC, particularly with the application of ddPCR. There was a recent study that focused on the response to targeted therapy in patients with metastatic CRC, and discovered that the methylation detection of neuropeptide Y (NPY) in blood by ddPCR could be useful to indicate the need for surgery, continuation of therapy, or disease progression according to its value change after primary treatment^42^. Further large-scale studies are needed to explore the potential role of ddPCR in the early monitoring of CRC patients after treatment.

Apart from being quantitative, the plasma volume used to generate ddPCR was much lower than conventional PCR. In our study, only 0.25 ml plasma per example was used by ddPCR, about 14-fold lower than the volume (at least 3.5 ml) required by commercially available Epi proColon SEPT9 assay^43^. Yu et al. compared MethyLight ddPCR with conventional MethyLight PCR in detecting DNA methylation for CRC and observed that the former had a 25-time lower limit of quantification (the lowest concentration of methylated DNA in a sample that could be detected) and a 20-time lower limit of detection (the lowest non-zero concentration in a sample with positive results) than the latter^37^. Pcardo et al. have also confirmed that droplet digital quantitative methylation-specific PCR (dd-QMSP) could detect a low amount of 0.025 ng DNA, tenfold lower than conventional QMSP on account of its high quality to detect reaction with light concentration^25^. The smaller volume of plasma samples required for ddPCR would enable repeated examinations possible and increase the potential clinical utilities, particularly as a screening and monitoring test.

This study also has some limitations. While this was an explorative feasibility study evaluating the role of ddPCR for SEPT9 methylation, the sample size of this study might limit further subgroup analysis of cancer patients according to different stages and tumor locations. As the frozen time of the plasma samples before detection was different, the DNA quality might be affected and impacted on the methylation results. Ideally, fresh plasma samples could be used to reduce this quality issue in future analysis. Additionally, with the current cut-off values of SEPT9 methylated ratio and abundance through the ROC curves, the sensitivities and specificities were not good enough as the commercially available Epi proColon 2.0 test (71.1–95.6% and 81.5–99% separately^20^. However, Epi proColon 1.0 test had a large range of sensitivity from 48.2 to 82% in detecting CRC^20^. The SEPT9 detected by the ddPCR system could be further refined with optimization of results. Besides, the methylation detected by ddPCR was only performed at 3-month after operation, more subsequent time points should be determined to assess the potential long-term monitoring role.

In conclusion, we have demonstrated the feasibility of using a low volume of plasma samples to detect methylated SEPT9 levels in CRC patients through ddPCR. Among various ways of expressing the levels of SEPT9 methylation, we found that SEPT9 methylated abundance performed better than the methylated ratio and concentration. The combination of SEPT9 methylated abundance with CEA was also found to have better performance to distinguish CRC patients than either alone. At 3-month after CRC operation, there was a significant decrease in the methylated abundance, especially in stage III and distal cancers. Further studies would be needed to evaluate and optimize the performance of this new approach of measuring SEPT9 through ddPCR.

## Supplementary Information

Supplementary Figures.
