# Supplementary material for: Application of droplet digital polymerase chain reaction of plasma methylated septin 9 on detection and early monitoring of colorectal cancer
Source: Sci Rep. 2021 Dec 6;11:23446. doi: 10.1038/s41598-021-02879-8 (PMC8648834; doi:10.1038/s41598-021-02879-8)
Supplement: Supplementary file 1 — Supplementary Figures. [file 41598_2021_2879_MOESM1_ESM.pdf]

Application of Droplet Digital Polymerase Chain Reaction of Plasma Methylated Septin 9 on Detection and Early Monitoring of Colorectal Cancer

Zhi Yao Ma, PhD[1]; Cherry Sze Yan Chan, MSc[1]; Kam Shing Lau, MMSc[1]; Lui Ng, PhD[2]; Yuen Yee Cheng, PhD[3]; and Wai K. Leung, MD[1]

[1]Department of Medicine, University of Hong Kong, Queen Mary Hospital, Hong Kong

[2]Department of Surgery, University of Hong Kong, Queen Mary Hospital, Hong Kong

[3]Asbestos Diseases Research Institute, Sydney Medical School, The University of Sydney

Corresponding author: Wai K. Leung, MD, Department of Medicine, University of Hong Kong, Queen Mary Hospital, Pokfulam Rd, Hong Kong; Phone No.: (852) 22553348; waikleung@hku.hk

>chr17: 77281451-77499029 (forward)

CAGAGAACTTTGTTTGGCTGCCCAAATACAGCCTCCTGCAGAAGGACCCTGCGCCCGGGGAAGGG GAGGAATCTCTTCCCCTCTGGGCGCCCGCCCTCCTCGCCATGGCCCGGCCTCCACATCCGCCCACAT CTGGCCGCAGCGGGGCGCCCGGGGGGAGGGGCTGAGGCC

Forward Primer: AGAGAAtTTTGTTTGGtTGtttAAATAtAG

Reverse Primer: AAAAAAAATTCCTCCCCTTCC

Methylated Probe: FAM-TGtAGAAGGAtttTGCGttCGG

Unmethylated Probe: HEX-tTGtAGAAGGAtttTGtGtttGG

Figure S1. Primers and probes of septin 9.

The forward and reverse primers were used to bind sequences (gray background) on two sides of human septin 9 methylated sites. The FAM- and HEX-labeled probes (underlined, cover two CpG sites) were designed to separately bind methylated and unmethylated CpG sites (red) in the DNA. Methylated cytosines would remain the same through bisulfite treatment.

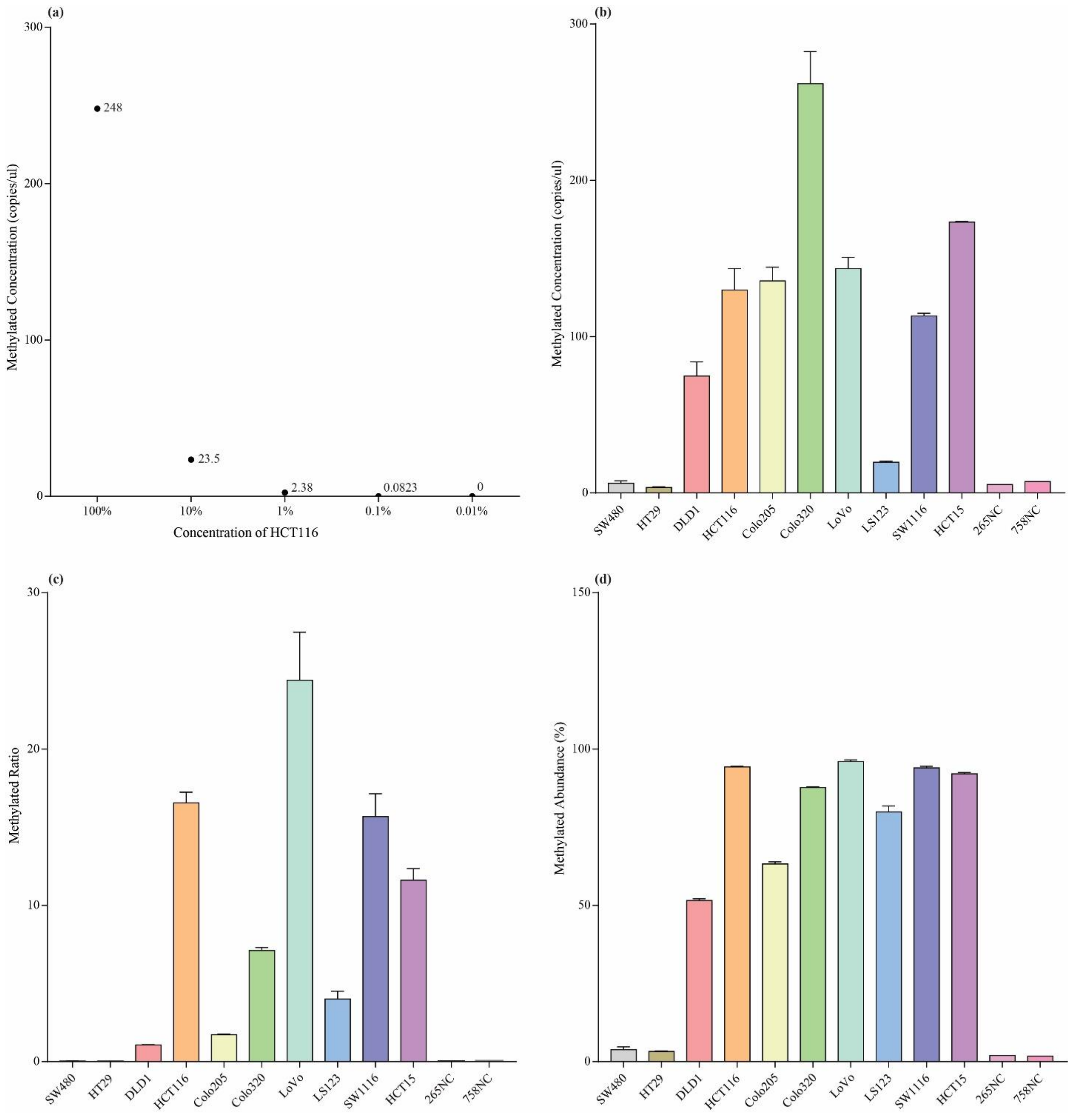

Figure S2. SEPT9 methylation detection in CRC cell lines and cancer-adjacent tissues.

(a) represented the dilution test of SEPT9 methylated concentration detected in decreasing concentration of HCT116 (from 100% to 0.01%). (b), (c) and (d) represented SEPT9 methylated concentration, ratio (methylated to unmethylated concentration/copies) and abundance (fraction of methylated concentration/copies in the total) for ten CRC cell lines (SW480, DLD1, HT29, HCT116, Colo205, Colo320, LoVo, LS123, SW1116 and HCT15) in triplicate PCR reaction and two cancer-adjacent normal tissues (265NC and 758NC) in single reaction.
